# Adapting an Adolescent and Young Adult Program Housed in a Quaternary Cancer Centre to a Regional Cancer Centre: Creating Equitable Access to Developmentally Tailored Support

**DOI:** 10.3390/curroncol31030095

**Published:** 2024-02-27

**Authors:** Marlie Smith, Simone Kurup, Kaviya Devaraja, Shaayini Shanawaz, Lorrie Reynolds, Jill Ross, Andrea Bezjak, Abha A. Gupta, Alisha Kassam

**Affiliations:** 1 Adolescent and Young Adult Program, Department of Supportive Care, Princess Margaret Cancer Centre, University of Toronto, Toronto, ON M5G 2C4, Canada; marlie.smith@uhn.ca (M.S.); kaviya.devaraja@mail.utoronto.ca (K.D.); sshanawaz@uwaterloo.ca (S.S.); abha.gupta@uhn.ca (A.A.G.); 2 Adolescent and Young Adult Program, Department of Supportive Care, Stronach Regional Cancer Centre, Southlake Regional Health Centre, Newmarket, ON L3Y 2P9, Canada; 3 Lawrence S. Bloomberg Faculty of Nursing, University of Toronto, Toronto, ON M5T 1P8, Canada; 4 Central Regional Cancer Program, Stronach Regional Cancer Centre, Southlake Regional Health Centre, Newmarket, ON L3Y 2P9, Canada; lreynolds@southlake.ca; 5 Childhood Cancer Care, Pediatric Oncology Group of Ontario, Toronto, ON M5G 1V2, Canada; jross@pogo.ca; 6 Department of Radiation Oncology, Princess Margaret Cancer Center, University of Toronto, Toronto, ON M5G 2C4, Canada; andrea.bezjak@rmp.uhn.ca; 7 Division of Medical Oncology and Hematology, Princess Margaret Cancer Center, University of Toronto, Toronto, ON M5G 2C4, Canada; 8 Department of Pediatrics, Southlake Regional Health Centre, Newmarket, ON L3Y 2P9, Canada; akassam@southlake.ca; 9 Division of Hematology/Oncology, The Hospital for Sick Children, Toronto, ON M5G 1X8, Canada; 10 Faculty of Medicine, University of Toronto, Toronto, ON M5S 1A8, Canada

**Keywords:** adolescent and young adult, oncology, cancer, program development, equitable access, supportive care

## Abstract

Adolescents and young adults (AYAs) with cancer, representing those between 15 and 39 years of age, face distinctive challenges balancing their life stage with the physical, emotional, and social impacts of a cancer diagnosis. These challenges include fertility concerns, disruptions to educational and occupational pursuits, issues related to body image and sexual health, and the need for age-appropriate psychosocial support within their communities. The Princess Margaret Cancer Centre (PM), a quaternary care center, established a specialized AYA program in 2014, offering holistic and developmentally tailored psychosocial support and currently, efforts are underway to expand this to other regions in the province to address the need for equitable access. The establishment process involves securing funding, conducting an environmental scan, identifying service gaps, developing clinical pathways, and implementing AYA supportive care. An accessible AYA program should also consider social determinants of health, social location, intersectionality, and an interdisciplinary health approach in understanding health inequities in AYA oncology care. This paper describes the processes implemented and challenges faced in creating a community-based AYA program beyond major resource-rich cities and efforts to address intersectionality.

## 1. Introduction

In North America, adolescent and young adult (AYA) is defined as including those between the ages of 15–39 years [1]. This period in life is marked by significant developmental milestones such as pursuing education or employment, establishing romantic and sexual relationships, family planning, and a deepened self-discovery [2]. There can be derailment in this development and health trajectories with the occurrence of a cancer diagnosis [3]. Although a cancer diagnosis inherently disrupts life for patients of all ages, cancer has an augmented impact on AYA patients, especially regarding fertility preservation, diagnosis and treatment of mental health concerns, a pause in education or career life goals, sexual health, and premature death [4,5,6]. In Canada, the current landscape of AYA care includes the delivery of oncology support at adult cancer centers, most of whom have limited resources or programming dedicated to the unique needs of this population [7]. For patients who live in rural communities or who identify with marginalized populations, the presence of and access to specialized AYA services within cancer care is further limited. Moreover, psychosocial care at large urban centers is often provided by hospital employees, whereas patients at community centres often rely on external psychosocial support in a fee for service model [8,9], further highlighting inequities.

AYA oncology programs must prioritize accessibility for patients of diverse backgrounds [10,11], acknowledging the importance of factors such as gender identity, sexuality, race/ethnicity, religion, socio-economic status, immigration status, and physical location. Additionally, consideration of historically oppressed members of the LGBTQ+, Indigenous, and Black communities, highlights the interconnectedness of social and personal identities with the behaviors and perceptions of patients [12]. Drawing on an intersectionality framework allows clinicians to remain cognizant of the compounding impact of an individual’s unique identity in their cancer care [13,14].

The purpose of this paper is to describe the development of an AYA oncology program at a community-based cancer center via a novel collaboration with an established program at a larger quaternary cancer center. A quaternary cancer center is a specialized healthcare facility that not only offers comprehensive cancer treatment and research services but also serves as a referral center, providing expertise in a complex and advanced cases, often involving cutting-edge therapies and experimental treatments. This program expansion pathway may serve as a pilot model to inform further expansion of AYA oncology care for patients independent of their jurisdictions. We also discuss the importance of recognizing diversity and the intersectionality of identity and program development. It is our hope that this paper offers unique insights into expanding AYA supportive care access and adapting the nature of the support offered to patients’ unique identities.

## 2. SDH and Intersectionality

### 2.1. Social Determinants of Health (SDH)

Social determinants of health are the conditions in which individuals are born, grow, live, work, and age, encompassing a range of economic, social, cultural, and environmental factors. These determinants play a crucial role in shaping health outcomes, influencing access to resources, opportunities, and services, and contributing to health inequities within populations [15]. The social determinants of health play a crucial role in shaping individual’s well-being, influencing factors such as access to education, economic opportunities, and healthcare, which collectively impact the overall health of general population [16]. Furthermore, disparities in social determinants can contribute to an increased risk of cancer among certain groups, highlighting the need for addressing social inequities to enhance cancer prevention and control efforts. Social determinants of health significantly impact the cancer experience by influencing factors such as access to timely and quality healthcare, economic opportunities, education, and social support, thereby contributing to disparities in cancer prevention, diagnosis, treatment, and overall health outcomes among diverse populations [17].

Marginalized groups such as Indigenous, LGBTQ+, and Black people have mistrust due to their perceptions of discrimination and racism in healthcare settings creating systemic barriers to routine screenings, potentially delaying diagnosis [18], and increasing the risk of being diagnosed with late-stage cancer with poorer prognoses [19]. Furthermore, the quality of life of marginalized people navigating a cancer experience is disproportionately impacted by their SDH. In addressing the unique needs of AYAs facing cancer, our focus is on modifying social determinants of health related to education, economic opportunities, and social support. By targeting these determinants, we aim to enhance AYAs’ access to tailored support, improve educational and vocational outcomes, and foster a supportive social environment, ultimately contributing to improved overall well-being and health outcomes during and after cancer treatment. With this knowledge, it is our responsibility to continue developing and expanding the AYA program as an intervention to address these systemic issues.

### 2.2. Intersectionality

Intersectionality (Crenshaw) serves as a framework in healthcare for understanding health inequities [20]. Although AYA cancer patients all share this “cancer patient” label, they also represent a group comprised of unique, compounding, and intersectional identities. Defining the AYA population solely in terms of age without accounting for cultural diversity can perpetuate implicit bias [12,18,20]. An effective and sustainable AYA program should not only meet patients’ medical and developmental needs but also meet needs based on their identity. Building a program with an awareness that social and personal identities are interconnected will help healthcare providers understand why patients and families perceive and cope with circumstances regarding their AYA cancer experience differently, and thus be more inclusive in their care [10,21]. For meaningful change to occur, there is a need for an awareness and prioritization of intersectionality across all stakeholders involved in cancer care, from researchers, direct practice clinicians, educators, policymakers, funders, and health organizations [22].

Social location refers to a person’s position in society based on socially constructed factors such as gender, class, education, employment, socioeconomic status, identity, geographic location, mental health, and disability [23]. Access to quality cancer care can be further impacted by individuals’ SDH [13], specifically, individuals’ social location may lead to inequities [24]. Both concepts can negatively impact patients’ health outcomes across the cancer continuum from routine screening to survivorship [25,26]. Health equity seeks to reduce inequalities and increase access to care that is conducive to health [24].

### 2.3. Interdisciplinary Health Approach

An interdisciplinary approach to AYA cancer care involves collaboration among healthcare professionals from various disciplines to address the unique physical, emotional, social, spiritual, and developmental needs of individuals in this age group who are diagnosed with cancer. The AYA stage of life is a time of self-discovery of their relationship with social constructs, such as religion, culture, and race. A multidisciplinary approach addresses the holistic needs of individuals in this age group, fostering improved treatment outcomes, quality of life, and long-term well-being [5,16,27]. This approach recognizes individuals’ unique identity and provides an opportunity to modify inequalities related to SDH. We refer our patients within PM to spiritual care practitioners on site; however, such a program is not in place at the Stronach Regional Cancer Centre at Southlake. It is our goal to build connections and local capacity with cultural and spiritual groups in the community to offer specialized spiritual care.

## 3. The Program Development Process

### 3.1. Development of the Princess Margaret Cancer Centre (PM) AYA Program

The PM is an example of a well-resourced quaternary care centre [28], and acts as a referral center for complex cases accepting local, national, and international patients. The AYA program is one of the many specialized services offered at PM [29] and was founded in 2014 after identifying a gap in care for this subpopulation. The program was developed by a medical oncologist with a special interest in the AYA population who then hired and trained a clinical nurse specialist (CNS) to execute the program [29]. The program was developed to optimize the supportive care for AYA patients through a biopsychosocial lens [30], incorporating biological, psychological, social, behavioral, and systemic processes impacted by a patient’s disease whilst highlighting factors influencing behaviors and perceptions [31,32].

The CNS conducts consultations with patients in the form of a 45 min phone call, video call, or in person appointment. The CNS conducts semi-structured consultations that are conversational in nature, discussing common documented concerns for AYAs navigating cancer. The consultation and its contents are dynamic in nature and ever changing to accommodate for the changing needs of AYAs and patient feedback. Currently, through these consultations, the CNS collaborates with patients and their family to identify their unmet needs and provides education on common concerns, including fertility, sexual health, fatigue, returning to work, and wellness. The CNS then arranged ongoing follow-up and triages to appropriate resources, both internally at PM and externally through community-based organizations [31,32,33]. The PM AYA program developed internal and external referral pathways to specialized clinics related to coping, fertility, mobility, survivorship, and peer connection. These referral pathways were established by the CNS ‘knocking’ on doors and identifying providers who have an interest/expertise in supportive care for the young person. These referral pathways continue to develop to meet the evolving needs of AYAs.

The CNS also has had additional training in sexual health [34], enabling her to offer counseling advocated by American Society of Clinical Oncology and Cancer Care Ontario [35,36]. Examples of sexual health counseling include assessment of sexual well-being, impotence, climacteric symptoms (in those with ovaries), and resulting impacts on relationships. Additionally, the CNS assesses a patient’s ability to engage in activities of daily living and can provide education regarding non-pharmacological techniques to manage fatigue and education for dietary modifications needed while undergoing treatment. In addition to providing direct clinical care to AYA patients, the CNS supports the professional development of frontline nurses and allied staff. By providing education sessions and just-in-time teaching opportunities, the CNS builds capacity for clinicians to engage in discussion regarding AYA patients’ unique and developmental needs, such as fertility, sexual health, and body image [37]. The CNS also collaboratively develops clinical pathways to meet the changing needs of AYA patients and engages in research to advance AYA oncology care in Canada.

Patients have reported that receiving AYA supportive care improves patient satisfaction with cancer information, social support, sexual health, fertility, physical appearance, and navigating work and school life [32].

### 3.2. Our Team

**Figure 1:**
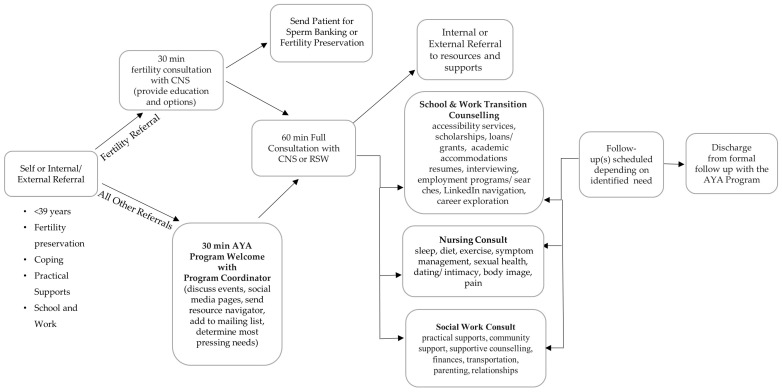
AYA referral pathway.

With continued professional development and recruitment of additional interdisciplinary team members; the current “domains” of AYA supportive care include fertility preservation, sexual health, spirituality, finances, school, work, symptom management, diet, exercise, sleep, relationships, parenting, coping, peer connection, and practical support. The flow of patients through the AYA program, including which actions are carried out by specific team members is shown in (Figure 1) [38].

Patients can access the AYA program through a provider or self-referral. A large focus of the program is facilitating peer connections within the AYA community through virtual meetups, virtual book club, yoga classes, cooking classes, art therapy, a strong social media presence, and in-person special events [29]. The discharge process for AYA from our program is thoughtfully tailored, with continuous assessment of the need for ongoing support and readiness for discharge during each patient interaction. While discharge signifies the conclusion of one-on-one clinical support from our social workers (SW) and clinical nurse specialists (CNS), the discharged patient maintains a connection with the AYA community and retains access to our services as necessary in the future. Our flexible approach allows patients to re-engage with the program at any time, ensuring ongoing support and assistance whenever needed.

An interdisciplinary AYA advisory committee was also created to expand the perspectives and expertise informing the program [8,29,31]. The advisory committee consists of psychologists, a pediatric nurse, adult and pediatric medical oncologists, a physician’s assistant, a music therapist, a PM hospital foundation representative, researchers, AYA palliative care team providers, and a patient representative [29]. Collaboration with community leaders in AYA oncology care and/or external clinical support services, cultural community groups, patient support and advocacy groups, researchers, and educators provide diverse perspectives and ensure a holistic understanding of the broader societal impact of the AYA program. The committee helps stimulate ongoing improvements to programming at various levels including direct practice, advocating for equitable funding allocation, and research and policy change with the AYA patient perspective at its centre. This patient representative shares personal experiences navigating cancer and helps validate service and research gaps and prioritize future initiatives.

## 4. Expansion of AYA Care to a Community Cancer Center

The PM Cancer Care Network developed a partnership with the Stronach Regional Cancer Centre (SRCC) at Southlake. In the form of a hub-and-spoke organization design, an opportunity arose to develop the first community based AYA program to address patient needs outside the Greater Toronto Area. Specifically, this model enables the delivery of assets, such as resources and specialized clinicians, to be primarily housed at an anchor establishment (hub) and enriches the services and support currently available at local cancer centres (spokes). Despite seeing roughly 100 patients per year at SRCC, we are still learning about the population’s characteristics and needs. This collaboration ensures that AYA patients can access comprehensive and tailored oncological support within their local community, bridging the gap between regional and quaternary care.

### 4.1. Securing Funding for Program Expansion

The first step of establishing an AYA program involves identifying a local AYA champion to be the program medical director (MeD). The MeD will then begin the engagement of top stakeholders, including hospital executives, followed by patient partners and existing supportive care personnel. The MeD will oversee the development of the everyday operations of the program, and importantly, lobby for funding to support hiring a clinical nurse specialist and the initial phases of program development. Initially, program funding may rely solely on philanthropy with gradual expansion to include hospital-based funding. This pivot requires the measurement of program impact and success and thus, measurement tools should be established upfront, even prior to program initiation [32,39]. The success of the PM AYA Oncology program is evident from initial evaluation efforts regarding patient satisfaction with information provided by primary oncology providers (POP) in crucial domains such as cancer information, social supports and school/work, and the incremental benefit of the AYA-dedicated team care. An added value was perceived in essential domains like school/work, social support, physical appearance, sexual health, and fertility from the team dedicated to AYA care [33]. Further, anecdotal testimonies from patients highlight the importance of an AYA program to foster a patient community, provide support for their developmental concerns (e.g., fertility and sexual health) and providing support for system navigation, self-advocacy, and empowerment. We will collect data at both the “hub” and “spokes” to ensure our efforts are addressing patients’ needs in different communities.

At PM, we were able to collect data on patient volumes as well as on the impact of program implementation on patient satisfaction, patients’ perception of the AYA program’s added value, and patients’ perception of the need for improvement in care delivery (i.e., fertility preservation) [32,40].

### 4.2. Conducting an Environmental Scan

Once funding for the new AYA program was secured and a CNS was hired, a formal needs assessment of the current state of AYA oncology care at the regional cancer center was conducted. This assessment was then paired with an environmental scan conducted by the CNS to analyze the organization’s external and internal environments impacting patient experiences at the regional cancer center. The environmental scan was conducted by drawing information from person sources, such as key hospital stakeholders and community partners, and non-person sources, such as databases and internet searches [41].

**Non-person sources.** A literature search was conducted to learn of AYA program structures within the province of Ontario and within Canada. AYA programs across Canada consist of some combination of the following members of an interdisciplinary team: A medical oncologist, a clinical nurse specialist, psychiatrists and social workers, researchers, a program coordinator, a school/work transitions counsellor, spiritual care, rehabilitation medicine, palliative care, and a radiation oncologist [10,31].

**Person sources.** Key hospital stakeholders included the cancer program director and medical director, the heads of medical and radiation oncology, the diversity and equity inclusion department, an indigenous navigator, members of the psychosocial oncology department, members of the information and technology department, unit managers and educators, and corporate communications. Summarizing the meetings led to the development of a list of key program priorities and existing resources available.

Key community partners included disease-specific community organizations as well as community rehabilitation services and fertility clinics. The CNS collated a list of recommended resources for common AYA concerns in the form of a “resource navigator” document to be distributed to every patient who meets with the AYA CNS. These resources include psychosocial support options, peer connection opportunities, sexual health, body image and fertility support, wellness and rehabilitation programs, work, school, and financial supports, and supports for the caregivers and children of AYA patients.

### 4.3. Identifying Service Gaps

The conducted environmental scan was useful in analyzing the cancer center’s existing internal and external resources that could be leveraged to provide fulsome AYA oncology care. Additionally, various service gaps were identified including inequitable access to developmentally tailored support for AYAs navigating cancer treatment and life after treatment across jurisdictions. Findings were presented to the MeD and the hospital executive wherein three initial program priorities were agreed upon: (a) to offer consistency in discussing fertility preservation and referring patients to specialized clinics, (b) to provide developmentally tailored psychosocial support for common AYA concerns such as sexual health and body image, and (c) to improve overall clinician education and recognition of unique AYA needs and to empower them to lead conversations related to developmental milestones and impact of cancer. A strategic plan for addressing these initial program priorities was developed in the form of a program logic model (Table 1).

The program logic model serves as a foundational framework for describing and evaluating the adaptation process of the AYA program by systematically addressing identified service gaps and establishing key priorities. It enables a structured approach to strategic planning, clearly defining objectives such as consistency in discussing fertility preservation, tailored psychosocial support, and enhanced clinician education. The model provides a roadmap for the implementation of these priorities, guiding the adaptation process to ensure a comprehensive and developmentally appropriate AYA oncology program.

### 4.4. Maintaining Connections to the Princess Margaret Cancer Centre AYA Program

A key component of the expansion of the AYA program to the regional cancer centre is a partnership with a larger cancer centre. This partnership is grounded in a hub and spoke model, whereby PM serves as the hub providing the core staff, programing, and resources to patients in the regional centre which is the spoke. PM provides training to the staff and providers at the spoke site, so there is continuity in understanding and best practices for AYA needs. Having this model as the framework for expanding the AYA program will ensure that a certain standard of care is met regardless of the capacity to support AYA patients internally and provides access to key specialized central resources associated with a resource rich centre.

### 4.5. Communication and Referrals

A seamless and inexpensive method of establishing communication and referrals involves establishment of a program email address such as aya@ [insert hospital domain]. Brochures and social media posts were also created to advertise the newly formed AYA program and circulated through a featured hospital wide communication. Clinical rounds were organized to educate clinicians and allied staff within each department at the cancer center on the uniqueness of AYA needs, to empower the staff to learn and increase comfort for these conversations, and to refer patients to the AYA program for additional support. Notably, an automated referral process was developed so that all new AYA patients will have the opportunity to meet the CNS at diagnosis, eliminating referral bias and practice variability, and addressing overall lack of AYA patient referrals to supportive care resources [42].

### 4.6. Evaluation and Intersectionality

Within 7 days of initial contact, patients are sent a patient satisfaction survey. The purpose of this evaluation is to display how our team is addressing the known service gaps experienced by AYAs navigating cancer. We hope to collect data that proves that our program meets the patient’s unique developmental needs and that our team provides information above what was provided by AYA patients’ primary oncology teams to identify areas of strength and areas that require attention. Additionally, the current hospital patient advisory committee is actively recruiting an AYA patient partner to help inform future program development. Meetings with the DEI team and Indigenous navigator were helpful in modifying screening tools (i.e., to include ‘moon cycle’ when enquiring about menses).

### 4.7. Challenged Faced

Through the development of our AYA program, we encountered several challenges, beginning with a notable lack of awareness of the specific needs of AYA patients. Establishing crucial connections within the community for support, creating pathways, and refining the referral system remain ongoing challenges. The collaborative development of consultations is hindered by gaps in services outside of the resource rich “hub”. This has required a focused approach to address, enhance, and standardize the support available to AYA patients across jurisdictions Moreover, securing sustainable funding is a critical hurdle that requires strategic planning for the program’s continued growth and success.

### 4.8. Future Initiatives

More work is needed to fill gaps in services. For example, the hospital does not have a cancer rehabilitation program and meetings have begun to strategize on how to offer rehab services to oncology patients. Moving forward, we will complement input from the feedback surveys and patient partner with focus groups to further address intersectionality for this community.

In addition, the unique palliative care needs for AYAs must be addressed. Currently, AYA patients at PM can be referred to the AYA Supportive Care clinic to address the physical and emotional symptom burden caused by dying as a young person. Specifically, this service helps patients find meaning in the face of a life-limiting illness and provides support for patients’ grief over the life they will never live. Our ongoing objective involves embedding a palliative care physician within our team to enhance the comprehensive and specialized care we provide our patients.

## 5. Discussion

This paper focuses on the formation of a partnership to enable the expansion of an AYA program from a resource-rich quaternary cancer center to a community-based cancer centre. We emphasize the importance of recognizing diversity and intersectionality in AYA care, considering factors such as gender identity, sexuality, race/ethnicity, religion, socio-economic status, immigration status, and physical location. The paper highlights the role of SDH and intersectionality in shaping an individuals’ cancer experience and influencing cancer risk among marginalized groups. Additionally, it discusses the interdisciplinary health approach in AYA cancer care, emphasizing the need for comprehensive support for young patients’ physical, emotional, social, and developmental needs. The program development process involves identifying service gaps, securing funding, conducting an environmental scan, and maintaining connections with a larger cancer center. Challenges faced include a lack of awareness about AYA patients’ specific needs, and future initiatives include addressing gaps in services, enhancing palliative care, and incorporating patient feedback and focus groups to address intersectionality within the community.

An accessible AYA program that considers all aspects of a patient’s social location in conjunction with their diagnosis can be crucial in preventative health measures [10]. We are modifying inequalities by bringing AYA cancer programming directly to patient communities which requires a local champion, funding, and engagement of stakeholders to move forward.

AYA oncology programming endeavors to reduce health inequalities including recognizing and addressing SDH, implementing initiatives that focus on promoting health, addressing challenges, and preventing diseases especially among marginalized populations and working collaboratively with communities, healthcare providers, and stakeholders to develop and implement strategies and programs that ensure equitable access to healthcare resources regardless of social location [24]. Developing AYA regional cancer programs provides an opportunity to modify these conditions, reducing these health inequalities, and helping to provide all AYA patients with the same opportunities within their cancer care.

To maximize our program’s impact and to ensure sustainability, stakeholders’ engagement beyond clinicians and service providers is needed. Specifically, engagement from policymakers and the government is imperative in securing funding to enable growth of our AYA program within Ontario and to extend the reach of our program. The government recognizes the importance of addressing health inequities; therefore, it is our duty to elevate the need for expanded AYA care to the policy agenda. This will enable equitable access to developmentally tailored support that recognizes patients’ intersectionality and will greatly improve outcomes for AYAs in Canada.

## 6. Conclusions

In conclusion, this paper outlines the development and expansion of an AYA oncology program, emphasizing the importance of addressing the unique needs of this population. The paper discusses the unique impact of a cancer diagnosis on AYA patients’ developmental milestones, particularly regarding future family planning, sexual health, mental health, education, and social disparities. Our paper highlights the benefit of a hub-and-spoke model to expand the support available to AYA patients outside of the Greater Toronto Area. We emphasize the significance of recognizing social determinants of health, incorporating intersectionality, and engaging diverse stakeholders to reduce health inequalities and ensure equitable access to tailored AYA care across jurisdictions. The expansion to a regional cancer center is described, addressing challenges, and outlining future initiatives to further enhance AYA oncology programming. Ultimately, the manuscript underscores the need for ongoing efforts, including policy engagement, to promote health equity and improve outcomes for AYAs in Canada.

## 7. Lessons Learned

Through the process of expanding the AYA program outside of Princess Margaret Cancer Centre we have learned the importance of environmental scans. The conducted environmental scan highlighted the resources that could be leveraged for the AYA program that were already embedded locally. This environmental scan also highlighted the importance of fostering relationships with stakeholders both within the partner hospital site and in the community. The scan was also fundamental in highlighting the gaps in services available to patients in their community. Further, the importance of prioritizing the recruitment of patient partners from diverse backgrounds and lived experiences is crucial in the adaptation of an AYA program to meet the needs of the local patient population. Our team endeavors to continue the efforts in recruiting eligible patient partners that are reflective of the local patient population that we serve. At this point, it appears that the fundamental needs of the local patient population are consistent with those of AYA patients in Toronto, indicating that we may have to adapt our approach as we move into more remote regions and the patient needs to diversify. A final lesson learned is the need for a flexible approach to psychosocial care and the need to continue adapting our approach and program offerings based on the everchanging social, emotional, cultural, spiritual, and physical development of the AYA population we serve.

## Figures and Tables

**Table 1:** AYA program logic model.

**What Is the Problem You Are Trying to Solve?**	There is a lack of developmentally tailored support and community programming for AYAs navigating cancer treatment and life after treatment outside of Toronto.
**Who Is Your Key Audience?**	AYA patients/families treated at cancer centres outside of Toronto. Clinicians and allied staff within cancer centres outside of Toronto. Local community support partners.
**What Is Your Entry Point to Reaching Your Key Audience?**	Accessible and streamlined referrals to the AYA program. Social media, advertising posters/brochures around the Cancer Centre Educate during designated rounds Feature in hospital communications
**What Steps are Needed to Bring About the Change?**	Develop urgent and routine referral processes to the AYA program Develop referral pathways for specialty service internally and externally (i.e., Fertility preservation) Prepare an asset map to utilize for referrals and resources Organize teaching sessions to all departments Prepare local resource navigator for patients to access Brainstorm patient event collaboration opportunities with community partners
**What Are the Measurable Effects of Your Work?**	Patient satisfaction (survey taken within 7 days of initial consultation with the AYA Program Staff) Number of patient referrals Patient attendance at events
**What Are the Wider Benefits of Your Work?**	Access to specialized and developmentally tailored support Access to patient community to reduce isolation Equitable access to care for AYA patients outside of Toronto Increase awareness of AYA needs and targeted approaches to needs
**What Is the long-Term Change You See as Your Goal?**	Increase fertility preservation rates for AYA Healthcare team recognition of unique AYA needs, and feels empowered to lean into conversation related to how AYAs’ cancer is impacting their life and developmental milestones All AYA patients outside of Toronto will have access to AYA specific support Positive patient satisfaction and value reports for the development and sustainability of the AYA program Patient reports of lower distress, reduced sense of isolation and increased sense of connection and community since AYA program involvement

## Data Availability

No new data was created or analyzed in this study. Data sharing is not applicable to this article.
